# Editorial: Recent highlights in the development of therapeutic antiviral strategies

**DOI:** 10.3389/fmicb.2023.1338999

**Published:** 2023-12-05

**Authors:** Cécile E. Malnou, Gaëtan Ligat

**Affiliations:** Institut Toulousain des Maladies Infectieuses et Inflammatoires (Infinity), Université de Toulouse, Institut national de la santé et de la recherche médicale (INSERM), Centre national de la recherche scientifique (CNRS), Université Toulouse III - Paul Sabatier (UPS), Toulouse, France

**Keywords:** antiviral strategies, viral inhibitor, vesicles, miRNA, genes editing and silencing

Recent developments in antiviral therapies are at the forefront of medical research, shaping the landscape of viral disease management and offering new hope in the fight against viral infections. Biotherapies, in the realm of antiviral treatments represent a cutting-edge approach to combat viral infections. These therapies leverage biological agents such as antibodies, interferons, or engineered immune cells to directly target viruses, or host factors, to improve the immune response. One notable example is the development of monoclonal antibodies, which have shown remarkable effectiveness in neutralizing viruses like SARS-CoV-2. The development of interferon-based therapies is another notable recent innovation. These treatments stimulate the host immune response, which improves its capacity to fight viral infections such as Hepatitis B Virus (HBV) infection. Recently, nucleic acid technologies have revolutionized antiviral strategies. mRNA vaccines, exemplified by those designed to combat COVID-19, have demonstrated effectiveness and adaptability against emerging viral variants. However, challenges persist in the pursuit of effective antiviral treatments. New therapeutic strategies need to be developed to overcome viral resistance. Beyond therapeutic strategies, a comprehensive approach to antiviral efforts includes a focus on prevention, early detection, and public education. Vaccination campaigns, innovative strategies for rapid viral detection, and public awareness programs all contribute to the fight against viral infections and improve global health outcomes.

In this Research Topic, we have collected original research and review articles covering many different aspects of therapeutic antiviral strategies.

Among them, several manuscripts mainly focused on antiviral therapies against coronaviruses. In an elegant study, Andreu et al. report for the first time the broad-spectrum antiviral activity of a Dextran sulfate-based extrapolymeric substance produced by the lactic acid bacterium *Leuconostoc mesenteroides* B512F. They evaluated the toxicity and antiviral efficacy upon inhalation of this exopolysaccharide substance in mouse models susceptible to SARS-CoV-2 infection, and demonstrated a strong inhibition of SARS-CoV-2 infection *in vivo*. Moreover, they also demonstrated a broad-spectrum antiviral activity of this substance against several enveloped viruses such as SARS-CoV-2, HCoV229E, HSV-1, in *in vitro* models and in human lung tissue. In another study, Piacentini et al. showed that the anti-infective drug nitazoxanide has a potent antiviral activity against three human seasonal coronaviruses HCoV-229E, -NL63, and OC43 in cell culture. Nitazoxanide does not affect HCoV adsorption, entry or uncoating, but acts at post-entry level, interfering with the spike glycoprotein maturation. Together, these two studies propose promising tools for the treatment of seasonal coronavirus infections. Finally, Guan et al. summarized the antiviral mechanisms of stress granules (SGs) and provided new insights into the development of SG-targeted antiviral drugs according to different pathways, particularly in the context of SARS-CoV-2.

Apart from the example of the study of Andreu et al. using a bacterium deriving molecule as antiviral treatment, other works presented in this Research Topic have used such antiviral approaches. In an interesting study, Wiggins et al. engineered two bacterial strains (*Lactobacillus casei* and *Lactococcus lactis*) for expressing scytovirin, a lectin deriving from cyanobacteria, on the bacterial surface. They demonstrated that both bacterial strains neutralize pseudotyped Ebolavirus in a cell-based assay. In a similar way, Kan et al. proposed a potential new drug to treat porcine epidemic diarrhea virus (PEDV) infection, by using *Lactiplantibacillus plantarum* supernatant. This acts by promoting the balance of intra- and extracellular Ca 2+ concentrations, thereby inhibiting PEDV proliferation with depends on intracellular Ca 2+ level.

In another work, Qi et al. evaluated the potential for synergistic effects of combination therapies to treat chronic hepatitis B infection. They presented two combination approaches aiming to target HBsAg and HBV-DNA. The first involved the use of antibodies followed by the administration of a therapeutic vaccine. The second combined antibodies with Entecavir. In both cases, they have shown that they constitute promising strategies to treat hepatitis B.

Finally, three reviews present current knowledge in antiviral approaches for chosen viruses. In the first one, Peng et al. presented functional epitopes and neutralizing antibodies of vaccinia virus, and discussed their potential value in the context of smallpox prevention and treatment. In a second manuscript, Afzal et al. reviewed current advances in therapeutic strategies, immune-based therapies and vaccine candidates for Hantavirus infections. Lastly, Gourin et al. presented the state of the current knowledge about anti-human cytomegalovirus (HCMV) therapies. They described the various molecules developed against HCMV with their mode of action, preclinical tests, clinical studies and possible resistance.

Significant progress has been made over the past decades in the development of new antiviral therapeutic approaches. In addition to the discovery and development of treatments for viral infections for which we do not yet have a strategy, the main challenge is to develop therapeutic approaches to overcome resistance mutations and toxicity of approved therapies. Overall, this Research Topic covered a large panel of antiviral strategies including new drugs, immune-based therapies, therapeutic antibody, and vaccine, and offer new perspectives for therapeutic intervention.

## Author contributions

CEM: Writing — original draft, Writing — review & editing. GL: Writing — original draft, Writing — review & editing.

